# Synthesis of Cellulose Nanoparticles from Ionic Liquid Solutions for Biomedical Applications

**DOI:** 10.3390/polym15020382

**Published:** 2023-01-11

**Authors:** Marta G. Fuster, Imane Moulefera, M. Noelia Muñoz, Mercedes G. Montalbán, Gloria Víllora

**Affiliations:** Chemical Engineering Department, Faculty of Chemistry, Regional Campus of International Excellence “Campus Mare Nostrum”, University of Murcia, 30071 Murcia, Spain

**Keywords:** cellulose, ionic liquid, nanoparticles, drug carriers, cell viability, HeLa, EA.hy926

## Abstract

A method for the synthesis of cellulose nanoparticles using the ionic liquid 1-ethyl-3-methylimidazolium acetate has been optimised. The use of a highly biocompatible biopolymer such as cellulose, together with the use of an ionic liquid, makes this method a promising way to obtain nanoparticles with good capability for drug carrying. The operating conditions of the synthesis have been optimised based on the average hydrodynamic diameter, the polydispersity index, determined by Dynamic Light Scattering (DLS) and the Z-potential, obtained by phase analysis light scattering (PALS), to obtain cellulose nanoparticles suitable for use in biomedicine. The obtained cellulose nanoparticles have been characterised by Fourier transform infrared spectroscopy (FTIR) with attenuated total reflectance (ATR), field emission scanning electron microscopy (FESEM) and thermogravimetric analysis (TGA/DTA). Finally, cell viability studies have been performed with a cancer cell line (HeLa) and with a healthy cell line (EA.hy926). These have shown that the cellulose nanoparticles obtained are not cytotoxic in the concentration range of the studied nanoparticles. The results obtained in this work constitute a starting point for future studies on the use of cellulose nanoparticles, synthesised from ionic liquids, for biomedical applications such as targeted drug release or controlled drug release.

## 1. Introduction

Nanoscience has developed in recent decades as a new branch of science that deals with the study, understanding and manipulation of materials at the nanometre scale. Nanotechnology, in turn, provides nanoscience with tools and technology, opening the door to new areas of study such as nanomedicine [1,2]. In this field, nanotechnology is expected to be a paradigm shift in the treatment of difficult-to-address diseases such as cancer, as conventional drugs have significant disadvantages, such as their toxic effect on healthy cells, as well as their low bioavailability [3]. The main advantages of using nanoparticles as drug nanocarriers are: (a) their protein-like size, (b) their high surface area/volume ratio, which allows the binding of different molecules by functionalising their surface, (c) their rapid uptake and controlled release, and (d) control over their surface and size during synthesis [4]. With all these features, new nanocarriers will be able to improve the release of poorly water-soluble drugs, increase selective release in target organs or tissues, and even improve disease diagnosis [5]. Among the challenges to be addressed in the development of new drug transporters are the control over morphology, size, and surface charge. For example, particle internalisation has been shown to be a function of nanoparticle size, shape and surface charge [6].

In addition, the growing concern for the environment implies the need to find synthesis mechanisms that minimise the use of pollutants and energy consumption. The use of cellulose, a biodegradable and biocompatible biopolymer [7,8], for the synthesis of nanoparticles, together with a synthesis procedure with low energy demand and the use of ionic liquids as solvents for the biopolymer [9], meets both the requirements of nanomedicine and the principles of green chemical engineering.

Cellulose is a polysaccharide whose molecular structure consists of D-glucopyranose units linked by β(1→4) glycosidic bonds that form a linear structure, in which hydrogen bonds form between oxygen atoms and OH groups between neighbouring chains and in the glucose chains themselves. These hydrogen bonds and Van der Waals forces aggregate the glucan chains, resulting in a stacking of microfibrils that constitute crystalline cellulose [10]. Four different polymorphs of cellulose are known, including cellulose I, II, III, and IV. In living plants, Cellulose I is the most widely distributed crystalline form, consisting of a combination of crystallites and amorphous disordered regions. Throughout the mercerisation process, the whole fibres are transformed into a swollen form and the assembly and orientation of the microfibrils are completely altered and the parallel chains of cellulose I are transformed into antiparallel chains of cellulose II [11]. Its empirical formula corresponds to (C_6_H_10_O5)_n_, where n is the degree of polymerisation, which can be extremely high. The network of hydrogen bonds in cellulose makes it a highly crystalline compound, insoluble in conventional organic solvents or water, and with a tendency to form aggregates [12]. Cellulose is the most abundant biopolymer on the planet, playing a fundamental role in forming structures both in the plant kingdom and in numerous microorganisms [10]. The wide availability of this material makes it a perfect candidate for the development of numerous applications without losing sight of economic and environmental sustainability [13,14]. Over the past two decades, nanocellulose has attracted a great deal of attention as a bio-based material in the engineering and research communities. Prospective applications of this raw material range from new types of composite to its application in medical devices and in the healthcare and pharmaceuticals industries [13]. Cellulose is difficult to hydrolyse or process because it cannot be easily dissolved or melted due to its intermolecular and intramolecular hydrogen bonds, high degree of crystallinity and its high level of polymerisation. A limited number of solvents have been used in the literature to dissolve cellulose. Zhao et al. [15] used N-methylmorpholine N-oxide (NMMO), Zhang et al. [16] used mixtures N-dimethylacetamide/lithium chloride (DMAC/LiCl), Jiang et al. [17] used mixtures dimethyl sulfoxide/paraformaldehyde (DMSO/PF), Tamai et al. [18] used mixtures 1,3-dimethyl-2-imidazolidinone/lithium chloride (DMI/LiCl), and Kuo and Lee [19] used NaOH/urea solutions. All these methods have a common drawback and that is the use of volatile and toxic solvents, the formation of by-products, process instability and, in general, unsafe and inefficient procedures.

Ionic liquids are molten salts that are liquid at or near room temperature and have great potential to easily dissolve cellulose [20]. These compounds are made up exclusively of ions, unlike molecular solvents such as water or organic solvents. The reason why these molten salts are liquid at room temperature is the lack of effective packing in their structure. In them, the presence of bulky asymmetric organic cations combined with relatively small inorganic anions and sometimes with organic anions generates lower melting points than those of common salts [21]. A fundamental characteristic of ionic liquids is their modulable nature and negligible vapour pressure, due to the weak Coulombic interaction between their ions, which hinders the formation of ionic pairs that lead to the volatilisation of the substance [22]. In addition to the above characteristics, ionic liquids exhibit high thermal and chemical stability.

Lee et al. [12] studied the ability of different ionic liquids to dissolve biopolymers. They highlighted 1-ethyl-3-methylimidazolium acetate, [emim^+^][CH_3_COO^−^], as one of the ionic liquids suitable for dissolving cellulose, thus replacing conventional techniques that required higher energy expenditure and the use of volatile solvents. In addition, other processes use 1-butyl-3-methylimidazolium chloride or 1-allyl-3-methylimidazolium chloride [23] for the dissolution of cellulose, which are solids at room temperature and much more viscous than [emim^+^][CH_3_COO^−^]. Vieira et al. reported the use of 3-dimethyl-amino-1-propylamine to dissolve the cellulose for producing bionanocomposites with tailored properties [24]. Swatloski et al. [25] also carried out experiments in which they prepared cellulose solutions in ionic liquids using microwaves, although an exhaustive study of the temperature reached during the process was not carried out. To our knowledge, there are very few studies on the synthesis of cellulose nanoparticles using ionic liquids. Han et al. [26] synthesised cellulose nanoparticles from microcrystalline cellulose dissolved in the ionic liquid 1-butyl-3-methylimidazolium chloride at 125 °C and using distilled water as antisolvent. After extensive washing and dialysis to remove the remaining ionic liquid, a high-pressure homogenisation step was necessary to improve the dispersion of the nanoparticles in water. Onkarappa et al. [14] obtained nanoparticles of different types of cellulose from solutions in the same ionic liquid. The chloride anion interacts through hydrogen bonding with the hydroxyl groups present in the cellulose, thus breaking the strong intermolecular hydrogen bonds that exist between the carbohydrate chains, favouring dissolution. Al Hakkak et al. [13] reported the synthesis and characterization of spherical cellulose nanoparticles using [emim^+^][CH_3_COO^−^], with diameters between 100 and 400 nm and with high uniformity.

Nanoparticles are of particular interest for drug delivery due to their high surface-to-size ratio, which makes them easily functionalisable and provides unique adsorption and controlled release properties of molecules [4]. The application of cellulose nanoparticles as drug carriers is of particular interest due to their biocompatibility, biodegradability, non-toxicity, and low cost, but studies on this biopolymer for this application are scarce [27] compared to others such as chitosan, dextran or silk fibroin [28,29,30,31,32]. Another advantage of cellulose nanoparticles is that the synthesis conditions can be varied to obtain nanoparticles of different sizes and surface areas, with different properties [33]. Some studies can be found on the use of cellulose nanoparticles as vehicles for drugs such as the antifungal drug griseofulvin [8], the antibiotics ciprofloxacin [34] or penicillin [35], betulinic acid for cancer treatment [36], omeprazole with anti-ulcer activity [37], or diclofenac [38]. There are also other studies on cellulose in the form of nanocrystals as drug carriers, e.g., curcumin [39] with chemotherapeutic effects. However, to date, no in vitro studies have been published on the feasibility of cellulose nanoparticles synthesised from ionic liquid for use in the biomedical field.

The main objective of the present work is the optimisation of the synthesis of cellulose nanoparticles using 1-ethyl-3-methyl imidazolium acetate, as well as the evaluation of their in vitro cytotoxicity to stablish their suitability for biomedical applications.

## 2. Materials and Methods

### 2.1. Materials

Microcrystalline cellulose was provided by Thermo Fischer Scientific (Molecular weight 370.3 g/moL, Polymerization Degree 193, >95% purity), referred to in this work as cellulose Thermo, and by Redwells, Co., Ward, UK (Molecular weight 324.3 g/moL, Polymerization Degree ≤ 300, >95% purity), referred to in this work as cellulose Redwells. The ionic liquid (95% purity), [emim^+^][CH_3_COO^−^], was purchased from IoliTec GmbH (Heilbronn, Germany), was vacuum-dried at 100 °C for 24 h, and used without further purification. All solvents and other chemicals were of analytical grade and were used without further purification. Purified water, obtained from an ultrapure water system (Millipore Direct-Q1, Billerica, MA, USA) with 18.2 MΩ·cm at 25 °C, was used throughout.

### 2.2. Synthesis of Cellulose Nanoparticles (CNPs)

**Scheme 1:**
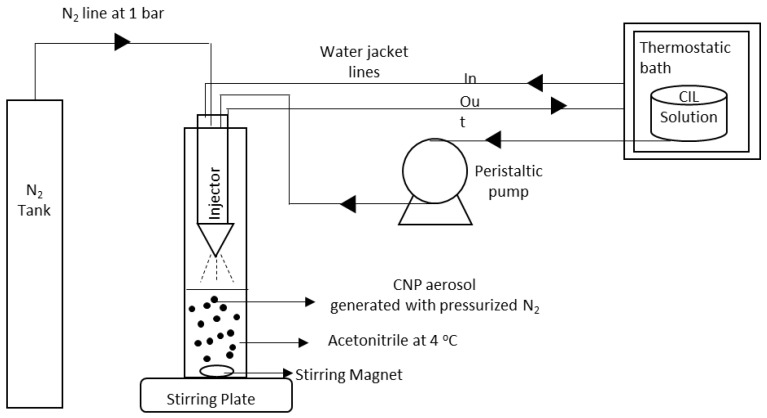
Experimental apparatus used for the CNPs synthesis process.

CNPs were obtained using the experimental setup described by Fuster et al. [30] to obtain silk fibroin nanoparticles, with modifications [40] as shown in Scheme 1.

First, a cellulose-[emim+][CH_3_COO^−^] (CIL) solution (2% wt.%) was obtained by mixing 3 g of cellulose with 150 g of [emim^+^][CH_3_COO^−^] in a round bottom flask under a nitrogen atmosphere at 80 °C for 16 h with magnetic stirring. The solution was loaded into a thermostatic bath at 60, 70 or 80 °C, then pumped and precipitated through a thermostatically controlled 0.7 mm two-fluid nozzle (from a Mini Spray Dryer B-290, BÜCHI Labortechnik, Flawil, Switzerland, Part No. 044698) into 50, 100, 150, or 200 mL of acetonitrile (4 °C) while stirring (1800–2200 rpm). The flow rate of the CIL solution in the spray was kept at 13.64 mL/min (Range 500, 12.5 rpm). The nitrogen pressure was continuously maintained at 1 bar during the whole process. After the precipitation process, the precipitated solution was agitated for two hours. After the synthesis was completed, the particles were washed extensively with water to remove the ionic liquid by centrifugation at 13,400 rpm. After the two washes, the particles were homogenised through a 3/8” conical horn of a Branson 450D sonicator (Emmerson Ultrasonic Corporation, Dansbury, CT, USA), with pulsed ultrasonic steps at an amplitude of 30% and resuspended in Milli-Q water. Aliquots were deposited in 90 mm × 14 mm Petri plates. After the samples were frozen overnight at −20 °C, the particles were placed in the freeze dryer (Thermo Scientific, Waltham, MA, USA) for 72 h at a temperature of −55 °C and 0.5 mbar to obtain dry particles. To recover the ionic liquid from the wash fractions, a BÜCHI RE-111 rotary evaporator, at 80 °C and 80 mbar, was performed. As previously mentioned, due to the very low vapour pressure of the ionic liquid, it can be separated from the acetonitrile, and both can be reused afterwards. The recycled ionic liquid was filtered through 0.22 µm diameter pores and then kept for 24 h in a vacuum oven at 100 °C, after which it was kept in a desiccator under vacuum and phosphorus pentoxide atmosphere until reuse. Table 1 summaries the conditions under which CNPs were synthesised and the codes with which they were labelled.

There are many processes described in the literature for obtaining CNPs [41], but most of them require a large number of dissolutions, precipitation filtration, centrifugation, and dialysis operations, and the subsequent concentration of the solution of CNPs obtained in the process, spending a lot of time and large amounts of water. Furthermore, the ionic liquids can be recycled and used without losing their properties in at least four successive cycles [42]. The process developed in this work has the advantage of requiring fewer steps, is a semi-continuous process and more scalable than those published in the literature.

### 2.3. Characterisation of Cellulose Nanoparticles

#### 2.3.1. Dynamic Light Scattering (DLS)

Once the CNPs were obtained, characterisation of the CNPs regarding their size was performed by DLS using a Malvern Zetasizer Nano ZSP (Malvern Instruments Ltd., Grovewood, UK), equipped with a laser of 4 mW power and a wavelength of 633 nm. The hydrodynamic intensity-weighted averaged diameter, called Z-average, and Z-potential were determined by DLS and phase analytical light scattering (PALS) techniques, respectively. After observing that CNPs did not show absorption at 633 nm under the measurement conditions, samples were prepared by suspending CNPs with a concentration of 1 mg/mL, using high-power ultrasonic energy at 30% amplitude for 1 min with 15 s pulses. The suspension was then transferred to a disposable capillary Z-potential measuring cell (DTS1070, Malvern). The data measured in a dynamic light scattering (DLS) experiment represent the correlation curve, which is a decaying exponential function for the scattering of single-sized particles. The correlation curve contains all the information regarding the scattering of particles within the sample being measured. By fitting the correlation curve with the exponential function, the diffusion coefficient (D) can be calculated and is proportional to the lifetime of the exponential decay. Knowing D, it is possible to calculate the hydrodynamic diameter, using a variation of the Stokes–Einstein equation. The backscattered light was measured at 173° relative to the source after 120 s of equilibration time at 25 °C. The size results shown are the average of 3 measurements, each consisting of 12 runs of 10 s with no delay between measurements. The Z-potential was calculated using the electrophoretic mobility of CNPs using the Henry equation, assuming the Smoluchoski approximation (κ_α_ = 1.5). The results for each Z-potential value are the result of the average of 6 measurements performed with a minimum of 12 runs. In addition, all Z-average and Z-potential measurements were performed in triplicate and the values were expressed as mean ± SD.

#### 2.3.2. Attenuated Total Reflectance Fourier Transformed Infrared Spectroscopy (ATR-FTIR)

Infrared spectral data of CNP-1F and CNP-2A and the microcrystalline cellulose from which they were derived, cellulose Thermo and cellulose Redwells, were obtained by attenuated total reflectance Fourier transform infrared spectroscopy (ATR-FTIR) on a Nicolet iS5 spectrometer coupled to a diamond crystal ATR iD7 module (Thermo Fischer Scientific, Waltham, MA, USA). OMNIC V9.9.471 software was used for spectral data control and processing. The interferograms were logged at a settlement of 4 cm^−1^ in the 4000–400 cm^−1^ spectrum range with a zero-fill factor of 2 and Fourier transformed using the Blackman–Harris 3-term apodization function. The average of each measured spectrum was derived from 64 scans. Before each measurement, an unsampled background spectrum was collected with the same number of scans. The spectra of the samples were acquired by loading 2 µL of the 0.66 mg/mL CNPs dispersion onto the ATR crystal after drying under a gentle stream of nitrogen. ATR-FTIR analysis was also used to study qualitative changes in crystallinity before and after the synthesis process for both starting materials. The focus was on changes in certain wavelengths that could be used to determine whether changes in crystallinity occurred after the dissolution and precipitation process of the cellulose in the antisolvent.

#### 2.3.3. Field Emission Scanning Electron Microscopy (FESEM)

The morphology of CNPs were characterised by FESEM, using a FEI Scios^TM^ microscope (Thermo Scientific, Waltham, MA, USA). A dispersion of 10 μg/mL CNPs was deposited on a silica support with a 2 nm thick platinum coating and dried with an infrared lamp.

#### 2.3.4. Thermogravimetric Analysis (TGA) and Differential Thermal Analysis (DTA)

The thermal behaviour of CNP-1F and CNP-2A nanoparticles was determined by TGA and compared with the thermal profile of the starting cellulose. The analysis was carried out with a thermogravimetric analyser (TA Instruments, SDT 2960, Waters LLC, New Castle, DE, USA), in a temperature range between 25 and 800 °C and with a heating rate of 10 °C/min under inert nitrogen atmosphere. The weight loss and temperature difference between the sample and an inert reference were recorded and plotted simultaneously as a function of temperature for TGA and DTA, respectively.

#### 2.3.5. In Vitro Cytotoxicity

Human cervical cancer cells (HeLa) and immortalised human umbilical cells (EA.hy926) were obtained from the American Type Culture Collection (ATCC, Manassas, VA, USA). The culture medium used was Dulbecco’s Modified Eagle Medium (DMEM) with a 4.5 g/L glucose content. The medium was supplemented with 10% (*v*/*v*) foetal bovine serum (FBS), 1 mM glutamax, 1% antibiotics (penicillin-streptomycin), and 1 mM pyruvate at 37 °C under a humid atmosphere containing 5% CO_2_ for both cell lines. The cells were subcultured with 0.25% trypsin-0.25 mM EDTA solution and the medium was changed twice a week. All cell lines were checked to be free of mycoplasma, before and after the experiments. A total of 5 × 10^3^ cells/well were seeded in a 96-well plate and incubated at 37 °C. After 24 h, the culture medium of each well was replaced with fresh medium and the cells were treated with different concentrations of CNPs (0–1 mg/mL). Growth medium without nanoparticles was used as a control. 48 h later, the medium was removed and Alamar Blue^®^ assay (Thermo Fisher Scientific, Waltham, MA, USA) was performed following the manufacturer’s protocol. Fluorescence was measured in a microplate reader FLUOstar Omega (BMG LABTECH GmbH, Freiburg, Germany) spectrophotometer using an excitation wavelength of 530 nm and an emission wavelength of 590 nm. Each sample was tested in at least three independent sets.

### 2.4. Statistical Analysis

GraphPad Prism 8.0.1 software (GraphPad Software, San Diego, CA, USA) was used to represent data as mean ± SD, calculated from at least three independent samples per condition. Statistical significance was determined by Tukey’s parametric tests (*p* < 0.05) and ANOVA for comparisons of two or more groups, respectively, given that normality (Kolmogorov–Smirnov, *p* > 0.05) and homoscedasticity (Levene, *p* > 0.05), (*p* < 0.05) were met (*p* < 0.05).

## 3. Results and Discussion

### 3.1. Characterisation of Cellulose Nanoparticles

#### 3.1.1. Dynamic Light Scattering (DLS)

**Figure 1:**
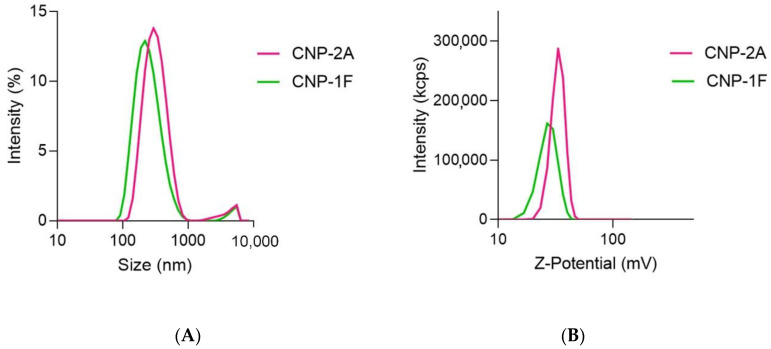
Particle size distribution (**A**) and Z-potential (**B**) of the CNP-2A and CNP-1F.

The synthesised CNPs were characterised by DLS to determine their hydrodynamic diameter (expressed as Z-Average), Z-potential, polydispersity index (PdI), and the average values of the peaks corresponding to the intensity, number and volume distributions (Table 2). In addition, Figure 1 presents the particle size distribution and Z-potential of CNP-1F and CNP 2A.

As can be seen in Table 2, when comparing the Z-Average values for samples CNP-1A, CNP-1B, CNP-1C, and CNP-1D, there was a tendency for the hydrodynamic diameter of CNPs to decrease as the volume of antisolvent increased in the range of 50 to 200 mL in the nanoprecipitation step, probably due to the influence in the the nucleation process of the particles in the antisolvent of the viscosity of the solution, the concentration and size of such droplets in the antisolvent. The Z-averages decrease from 537.4 to 274.9 nm. A trend of decreasing values of the hydrodynamic diameter of the nanoparticles with increasing nanoprecipitation temperature between 60 and 80 °C was also observed, mainly due to the influence of the viscosity of the solution in the formation of the droplets coming out of the spray. This can be verified by comparing samples CNP-1D, CNP-1E, and CNP-1F, whose Z-Average decreases from 274.9 to 233.4 nm. Finally, cellulose Thermo, provided by ThermoScientific, was found to provide smaller hydrodynamic diameter sizes in CNPs than cellulose Redwells, provided by Redwells, Co., UK, as can be seen by comparing samples CNP-1F (cellulose Thermo) and CNP-2A (cellulose Redwells).

Moreover, a polydispersity index (PdI) of 0.215 for CNP-1F can be considered acceptable, indicating a monodisperse size distribution, and the Z-potential, around 30 mV, is high enough to expect good a stability of the CNPs suspension (see Figure 1).

#### 3.1.2. Attenuated Total Reflectance Fourier Transformed Infrared Spectroscopy (ATR-FTIR)

**Figure 2:**
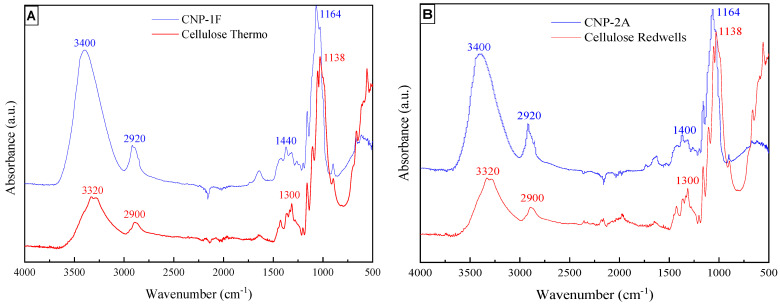
ATR−FTIR spectra of (**A**) CNP−1F and cellulose Thermo, (**B**) CNP−2A and cellulose Redwells.

The ATR-FTIR spectra of the CNP-1F and CNP-2A and of the microcrystalline cellulose from which they were obtained, cellulose Thermo and cellulose Redwells, respectively, were used for comparison and characterisation (Figure 2).

All four types of cellulose show broad absorption peaks [43], between 3320 and 3400 cm^−1^, assigned to hydrogen-bound -OH stretching vibrations. Peaks between 2900 to 2920 cm^−1^ are associated with C-H stretching vibrations. In addition, the peaks near 1640 cm^−1^ are attributed to the H-O-H deformation vibration of the absorbed water and the C = O conjugate stretching vibration. The absorbing band between 1300 and 1440 cm^−1^ corresponds to the asymmetric deformation of C-H. The peaks around 1038 and 1164 cm^−1^ are attributed to an O-H association band [14] and finally, peaks around 900 cm^−1^ correspond to β (1→4) glycosidic bonds present in cellulose [24]. No new bands are observed, suggesting that no significant chemical modification of the cellulose has occurred by the process of nanoparticles formation. Nevertheless, a slight shift of the absorption peaks of the CNPs towards higher wavenumbers with respect to the peaks of the microcrystalline cellulose is observed, suggesting a transition between the two crystalline forms of cellulose, from cellulose I to cellulose II [44,45,46].

As mentioned above, changes in crystallinity and hydrogen bond intensity can also be assessed by ATR-FTIR [47]. IR radiation is attracted by the molecules and induces vibrational motion; this vibrational movement in the cellulose molecule is affected by inter- and intramolecular interactions, especially the hydrogen bond. The cellulose polymer chain vibrates differently in well-ordered crystalline and less ordered amorphous phases [48]. Therefore, it is possible to assign related absorption bands to crystalline and amorphous regions. O’Connor et al. (1958) [49] established that the absorption band at 1429 cm^−1^ was typical of the crystalline regions of the polymer and that the absorption band at 893 cm^−1^ was characteristic of the amorphous regions. The ratio of the two bands was identified as a “crystallinity index”, later referred to as the “lateral order index (LOI)”. Subsequently, Nelson and O’Connor (1964) [50] defined another crystallinity index from the ratio of the absorption bands at 1372 cm^−1^ and 2900 cm^−1^, the so-called “total crystallinity index” (TCI). In the cellulose structure, three hydroxyl groups interact with other hydroxyl groups, forming valence bonds. This network of hydrogen bonds plays an important role with respect to crystallinity and chain structure. The cellulose II network is more compact than the cellulose I network, and the cellulose molecules are more closely interconnected, so the evaluation of the hydroxyl absorption bands is important for estimating the crystallinity of cellulose II fibres. Nada et al. [44] inserted an empirical relationship, called hydrogen bonding intensity (HBI), which provides a comparison of the ratio of the absorption bands at 3336 cm^−1^ and 1336 cm^−1^, closely correlated with the well-ordered crystal layer and the degree of in-termolecular regularity. The values for TCI, LOI and HBI for CNP-1F and CNP-2A and for the microcrystalline cellulose from which they were obtained, cellulose 1 and cellulose 2, are shown in Table 3, where no significant differences were found between the samples for all the indices studied (TCI, LOI, HBI), except for the CNP-1F nanoparticles of cellulose 1 that presented a lower TCI value and a significantly higher HBI. This observation suggests that CNP-1F cellulose nanoparticles are significantly less crystalline than the rest and that there are more hydroxyl groups available on these nanoparticles capable of interacting via inter- and/or intramolecular hydrogen bonds. On the other hand, it was observed that the symmetric absorption of CH_2_ bending at 1430 cm^−1^ decreased due to a reduction in the degree of crystallinity of CNP-1F [14], suggesting that the spherical cellulose particles contained more amorphous cellulose than the starting material. These results are in agreement with those provided by Al Hakkak et al. [13].

#### 3.1.3. Field Emission Scanning Electron Microscopy (FESEM)

**Figure 3:**
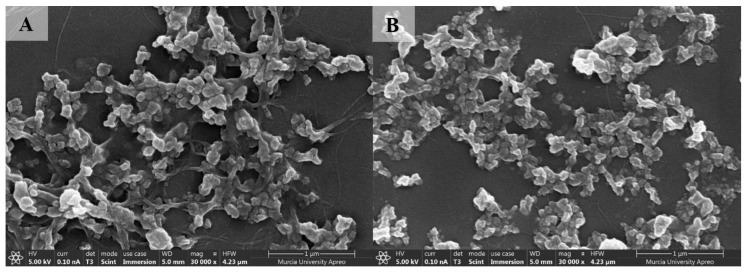

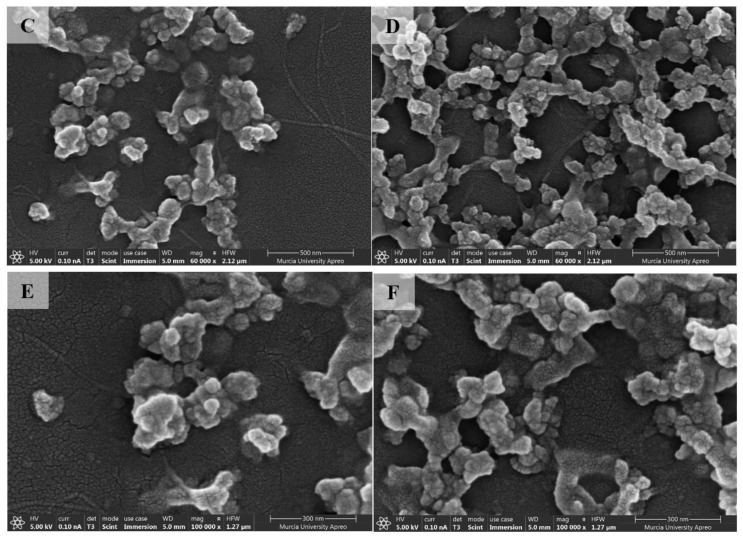
FESEM picture of (**A**) CNP-2A, (**B**) CNP-1F (30,000×), (**C**) CNP-2A, (**D**) CNP-1F (60,000×) and (**E**) CNP-2A, (**F**) CNP-1F (100,000×).

The morphology and size of the CNPs were evaluated by FESEM. Image analysis of cellulose nanoparticles synthesised by the optimised method is shown below for nanoparticles of cellulose Thermo (CNP-1F) and cellulose Redwells (CNP-2A). Images are shown in Figure 3 at 30,000× (A,B), at 60,000× (C,D) and at 100,000× (E,F).

The images show that the morphology of the CNP-1F and CNP-2A nanoparticles is granular, almost spheroidal. In addition, magnified images at 60,000× and 100,000× show that each particle is made up of aggregates of smaller particles, with irregular shape.

To compare the size observed by microscopy with that measured by DLS, the swelling effect that the particles undergo in the DLS measurement must be taken into consideration. This effect is because the nanoparticles interact with the water in which they are dispersed. In contrast, in the measurement by FESEM, the particles are dry dispersed, and this swelling effect does not occur [30]. In addition, the size measured by DLS includes the diffusion layer around the nanoparticle [51], which makes the sizes provided by DLS larger than those obtained by FESEM. This effect has been previously described by other authors [11,33,35]. Figure 3 shows that CNP-2A (Figure 3A) have a diameter of approximately 160 nm, while the CNP-1F (Figure 3B) have a smaller diameter of about 110 nm.

#### 3.1.4. Thermogravimetric Analysis (TGA) and Differential Thermal Analysis (DTA)

**Figure 4:**
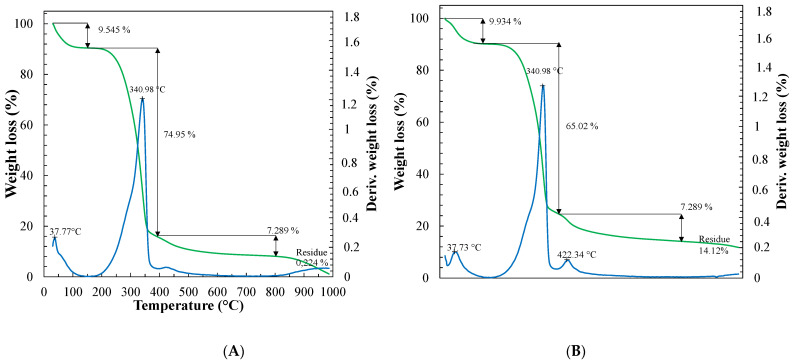
TGA (green) and DTA (blue) of: (**A**) CNP-1F and (**B**) CNP-2A.

The obtained cellulose nanoparticles (CNP-1F and CNP-2A) were characterised by thermogravimetric analysis (TGA) and differential thermal analysis (DTA) to study their thermal stability. The results are shown in Figure 4.

As can be seen, the curves obtained for both types of nanoparticles are very similar. Moisture evaporation in both CNP samples leads to weight loss at 100 °C. The next weight loss corresponds to the degradation of the cellulose, which in both cases takes place in two stages, presenting a first higher peak at a temperature of 341 °C and a second lower peak at a temperature of about 420 °C. This same two-step decomposition behaviour was observed by Han et al. [26] although these authors found the peaks at much lower temperatures (254.7 and 328.4 °C) indicating a lower thermal stability of their nanoparticles.

However, other authors such as Onkarappa et al. [14] state that the decomposition of cellulose nanoparticles takes place in a single stage, although at a temperature also significantly lower than that found in this work, again indicating that these nanoparticles are less thermally stable than those obtained in the present study.

#### 3.1.5. In Vitro Cytotoxicity

**Figure 5:**
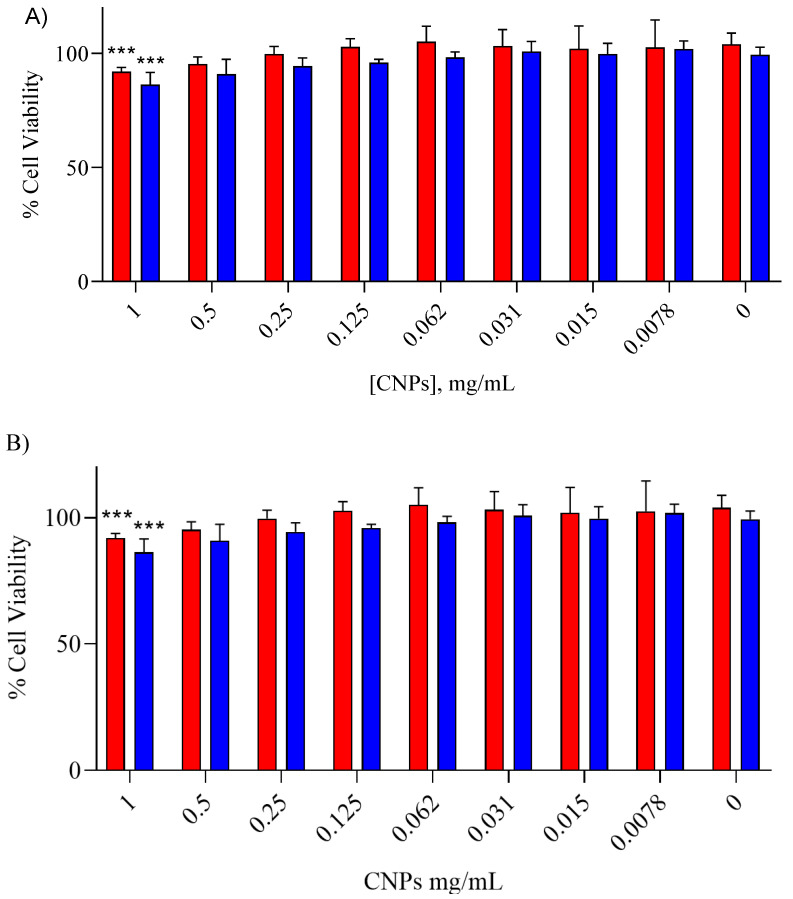
Cell viability assays in (**A**) HeLa and (**B**) EA.hy926, treated with CNP-1F (red) and CNP-2A (blue). The results shown represent 4 replicates per experiment. In both cell lines, untreated cells were used as controls. Values were compared using one-way ANOVA and express percent viability ± SD versus nanoparticle concentration. *** indicates *p* < 0.001, compared to control.

To evaluate the cytotoxic effects of the cellulose nanoparticles, CNP-1F and CNP-2A, and determine their suitability for biomedical applications, cell viability assays were performed with Alamar blue. The reaction is based on a dye, the redox indicator resazurin, which produces a colorimetric change and fluorescent signal in response to metabolic activity as well as cell survival and proliferation. Assays were performed on both a human cancer cell line (HeLa) and a healthy human cell line (EA.hy926). The results are shown in Figure 5.

As can be seen in Figure 4, for both cell lines the cell viability is higher than 85%, demonstrating the non-toxicity of CNPs to both cancer and healthy tissue cells [52]. Therefore, these results constitute a starting point for future studies on the use of CNPs synthesised from ionic liquids for biomedical applications as a drug release vehicle.

## 4. Conclusions

In the present study, two microcrystalline celluloses were used to prepare CNPs from the ionic liquid [emim^+^][CH_3_COO^−^]. It has been found that by increasing the volume of acetonitrile as antisolvent, and by increasing temperature in the precipitation step, the hydrodynamic diameter of the CNPs decreases significantly.

The FTIR spectra reveal that the CNPs show the same characteristic peaks as cellulose. No new bands are observed in the CNPs with respect to the preceding microcrystalline cellulose, suggesting no further chemical modification of the cellulose by the process. The FESEM images show a granular, almost spheroidal morphology, smaller in size than that measured by DLS due to the swelling effect that occurs in the suspended particles when using the latter technique.

TGA/DTA analysis shows that CNPs reveal a small weight loss around 100 °C, which is associated with the evaporation of bound water in the cellulose samples. The degradation of CNPs occurs in two stages, the main mass loss occurring at 341 °C, the second around 420 °C, as shown in the DTA curve. All CNPs have a good thermal stability.

Finally, CNP-1F and CNP-2A have been shown to exhibit no cytotoxic effects when assessing cell viability in both a human cancer cell line (HeLa) and a healthy human cell line (EA.hy926), making them suitable for use in biomedical applications. Moreover, due to the surface properties of CNPs, these results offer the possibility of their surface functionalization, which has a great potential for loading different types of drugs and applications.

## Figures and Tables

**Table 1:** Codes for the procedures assayed.

Nanoparticle Identifier Code	Cellulose Type	Precipitation Temperature (°C)	Antisolvent Volume (mL)
CNP-1A	Cellulose Thermo	60	50
CNP-1B	Cellulose Thermo	60	100
CNP-1C	Cellulose Thermo	60	150
CNP-1D	Cellulose Thermo	60	200
CNP-1E	Cellulose Thermo	70	200
CNP-1F	Cellulose Thermo	80	200
CNP-2A	Cellulose Redwells	80	200

**Table 2:** Particle size distribution and Z-potential of CNPs by DLS.

Nanoparticle Identifier Code	Z-Average (nm)	PDI	Peak Intensity (nm)	Peak Number (nm)	Peak Volume (nm)	Z-Potential (mV)
CNP-1A	537.4 ± 11.9	0.383 ± 0.006	830.5 ± 104.9	223.2 ± 60.4	897.5 ± 34.8	25.6 ± 0.5
CNP-1B	324.1 ± 7.8	0.255 ± 0.030	522.3 ± 27.6	239.6 ± 6.3	526.6 ± 18.0	34.0 ± 0.3
CNP-1C	332.9 ± 4.8	0.233 ± 0.005	480.1 ± 75.6	197 ± 58.1	507.1 ± 56.5	27.8 ± 1.4
CNP-1D	274.9 ± 5.3	0.212 ± 0.044	409.2 ± 93.9	194.8 ± 7.69	414.8 ± 87.0	32.1 ± 1.0
CNP-1E	271.5 ± 3.8	0.249 ± 0.017	482.2 ± 48.8	186.7 ± 4.2	480.7 ± 39.0	28.8 ± 0.6
CNP-1F	233.4 ± 6.0	0.215 ± 0.025	381.7 ± 110.6	157 ± 7.4	383 ± 83.0	28.5 ± 0.6
CNP-2A	310.0 ± 6.5	0.218 ± 0.026	496.5 ± 107.3	230.2 ± 7.6	492.6 ± 78.2	32.9 ± 0.3

Data are shown as mean value ± SD (standard deviation).

**Table 3:** Comparison of crystallinity indices and hydrogen bond intensity for CNP-1F, CNP-2A, Cellulose 1 and Cellulose 2.

Cellulose Sample	TCI	LOI	HBI
Cellulose 1	1.188 ± 0.0061 (1372/2900)	0.518 ± 0.0051 (1429/897)	1.223 ± 0.0093 (3338/1334)
Cellulose 2	1.155 ± 0.0050 (1372/2900)	0.527 ± 0.0060 (1429/897)	1.282 ± 0.0095 (3338/1334)
CNP-1F	0.617 ± 0.0108 (1364/2892)	0.413 ± 0.0048 (1418/894)	3.279 ± 0.0512 (3336/1336)
CNP-2A	0.958 ± 0.0018 (1364/2892)	1.003 ± 0.0062 (1418/894)	3.769 ± 0.0257 (3336/1336)

Data are shown as mean value ± SD (standard deviation).

## Data Availability

Data presented in this study are available on request from the corresponding author.
